# Non-Covalent Assembly of Multiple Fluorophores in Edible Protein/Lipid Hydrogels for Applications in Multi-Step Light Harvesting and White-Light Emission

**DOI:** 10.3390/molecules28166028

**Published:** 2023-08-12

**Authors:** Jingwen Ding, Challa V. Kumar

**Affiliations:** Department of Chemistry, University of Connecticut, Storrs, CT 06269, USA

**Keywords:** bovine serum albumin, decanoic acid, hoechst 33258, fluoresceine, rhodamine, coumarin, circular dichroism, SDS–PAGE, emission, absorption

## Abstract

The design and production of biodegradable and sustainable non-toxic materials for solar-energy harvesting and conversion is a significant challenge. Here, our goal was to report the preparation of novel protein/lipid hydrogels and demonstrate their utility in two orthogonal fundamental studies—light harvesting and white-light emission. Our hydrogels contained up to 90% water, while also being self-standing and injectable with a syringe. In one application, we loaded these hydrogels with suitable organic donor-acceptor dyes and demonstrated the energy-transfer cascade among four different dyes, with the most red-emitting dye as the energy destination. We hypothesized that the dyes were embedded in the protein/lipid phase away from the water pools as monomeric entities and that the excitation of any of the four dyes resulted in intense emission from the lowest-energy acceptor. In contrast to the energy-transfer cascade, we demonstrate the use of these gels to form a white-light-emitting hydrogel dye assembly, in which excitation migration is severely constrained. By restricting the dye-to-dye energy transfer, the blue, green, and red dyes emit at their respective wavelengths, thereby producing the composite white-light emission. The CIE color coordinates of the emission were 0.336 and 0.339—nearly pure white-light emission. Thus, two related studies with opposite requirements could be accommodated in the same hydrogel, which was made from edible ingredients by a simple method. These gels are biodegradable when released into the environment, sustainable, and may be of interest for energy applications.

## 1. Introduction 

The design of soft materials that are amenable for multiple energy applications—for example, light harvesting or white-light emission—is of current interest. Here, we describe a dual-purpose protein/lipid hydrogel that can be tailored for either light harvesting or white-light emission. Different systems are being developed to construct light-harvesting devices that harness a wider portion of the solar spectrum [1,2,3,4]. However, these systems still have fundamental challenges to overcome in terms of efficiency and the width of the solar spectrum that is harvested [5]. For example, solution systems are not suitable as coating materials, and solid-phase systems might inhibit energy transfer among the harvesting dyes unless they are separated sufficiently to avoid self-quenching but remain close enough for excitation exchange. Light harvesting over a wide wavelength range often requires multiple jumper dyes that participate in efficient excitation transfer to the active chromophore. Therefore, a light-harvesting device that converts blue photons to red photons and operates in the solid phase would be attractive. The design of such a device remains a current challenge [6].

An efficient two-step light antenna, using inorganic layered materials and organic dyes via non-covalent assembly in an aqueous suspension, was reported earlier [7]. Long-distance excitation migration among Ru(II) metal complexes in frozen matrices was also reported from our laboratory [8]. More recently, the self-assembly of protein-DNA complexes that are incorporated with up to four organic dyes indicated efficient energy transfer to the terminal acceptor [9]. Numerous examples of two- or three-dye light-harvesting systems are known, including supramolecular organogels made with impressive synthetic methods [10,11]; however, increasing the dyes to achieve a four-dye system, to cover a wider absorption spectrum, is rare.

In addition to the above requirements, light-harvesting systems are expected to have certain other characteristics, including being non-toxic, environmentally benign, sustainable, biodegradable on release into the environment, recyclable, and even self-healing. They are to be made from earth-abundant elements with simple preparation methods that can be used under resource-limiting conditions, preferably via non-covalent assembly. These challenges are addressed here, with a long-term hope of solving one or more of these issues.

The second application of these novel gels, demonstrated here, is white-light emission. These systems require that dye-to-dye energy transfer be restricted, which is opposite to the requirements of the light-harvesting systems discussed above. When dye-to-dye energy transfer is disengaged, emission from blue, green, and red dyes can simulate white-light emission. Thus, both of these strategies require control of dye-to-dye communication and dye sequestration in the matrix, without aggregation. The hydrogels reported here satisfy both of these divergent requirements in a single material, which is a unique advantage. 

There are a few main approaches to synthesizing white-light emitters. One is based on the inorganic metal complexes from ions such as Al^3+^, Zn^2+^, Ce^3+^, and Eu^3+^ [12,13,14,15], coordinated to suitable ligands [16,17,18]. These materials have some disadvantages, such as harsh experimental conditions for synthesis [19], and they may use hazardous chemicals [20]. Numerous examples consisting of two or more chromophores, usually covalently linked or loaded in layers or organic hydrogels, are studied extensively [11,12,21]. However, many of these materials are not environmentally friendly or are not suitable for LED coatings. 

Yet another approach to making white-light emitters is a bio-hybridized polymer material synthesized from salmon DNA [22]. Silk proteins were also used with a series of quantum dots to fabricate white-light emitters [23]. These approaches overcame some of the key problems of design, to a certain degree. However, there is still a big challenge in generating a low-cost, easy-synthesis, biodegradable, and non-toxic white-light emitter. 

Here, we designed and developed a protein/lipid hydrogel system as a platform to assemble light-harvesting systems or white-light-emitter coatings, with a dual purpose. Bovine serum albumin (BSA), a waste product from the meat industry, was chosen for these studies, due to its abundant availability and its biodegradability into nutrients when released into the environment. BSA is known to form hydrogels consisting of microscopic pores by heating, pH denaturation, or chemical crosslinking [24]. 

Decanoic acid (C10), a food ingredient, was used as the lipid in our studies, due to its availability in large quantities, its low cost, and previous studies from our laboratory that showed that it can protect proteins from thermal denaturation [25]. Above its critical micellar concentration, C10 is known to form micelles or even unilamellar vesicles [26]. We utilized these two components to form hydrogels that contain both the protein matrix and a lipid component, to facilitate the dissolution of a variety of organic dyes that may not otherwise have dissolved in aqueous solutions. We wondered if the C10 micelles could be loaded into the microstructure of BSA and sequester donor–acceptor pairs within these organized compartments for enhanced energy transfer and decreased oxygen quenching.

**Scheme 1:**
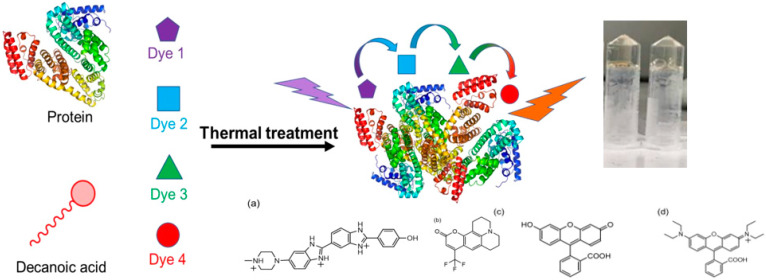
A physical mixture of BSA, decanoic acid, and selected dyes heated to quickly form a transparent, injectable, self-standing hydrogel (>90% water). Concentrations of multiple dyes were optimized for a high efficiency FRET or white-light emission, as desired. Chemical structures of dyes used were (**a**) Hoechst 33258, (**b**) coumarin 540A, (**c**) fluorescein, and (**d**) rhodamine B. These were selected based on their absorbance and fluorescence characteristics, as explained in the text.

Free-standing protein/lipid hydrogels were prepared by heating a mixture of the protein and the lipid, in the absence or presence of dyes (Scheme 1). By simply dissolving specific fluorescent dyes at appropriate concentrations in BSA/C10 solution prior to gelation, gels that showed efficient fluorescence resonance energy transfer (FRET) or white light emission were generated. For FRET application, the excitation of the donor at 350 nm resulted in an intense red emission at 590 nm (Scheme 1). We surmised that H and C, C and F, and F and R formed efficient donor-acceptor pairs, based on the overlap of the acceptor-absorption spectrum with the corresponding donor-emission spectrum. Thus, an energy-transfer cascade chain was envisioned with these four dyes. 

By inserting appropriate fluorophores, the above protein/lipid hydrogels were also turned into white-light emitters. Coating on a blue-emitting LED (365 excitations) turned it into a white-light emitting LED. The method developed here to prepare the hydrogels was robust, facile, and simple. These materials were based on components that were biocompatible, biodegradable on release into the environment, non-toxic, environmentally friendly, and low-cost.

## 2. Results and Discussion 

A novel kind of protein/lipid hydrogel platform was developed here that was amenable for divergent applications such as a light-harvesting system or a white-light emitter that demand different requirements for housing the chromophores. The hydrogel was composed of a common protein and a medium chain fatty acid, which were self-assembled without any chemical crosslinking. We chose bovine serum albumin (BSA) as the protein, due to its abundance and low cost. Upon heating, it is known to form hydrogels consisting of microscopic cubicles of tens of microns in size [27,28]. Our previous work also demonstrated a slow diffusion of oxygen within the protein hydrogels. However, most of these gels were opaque and not readily amenable for spectroscopic studies. We chose to add a medium-chain lipid to the protein solution to improve the clarity of the gels. On heating, a mixture of BSA and decanoic acid (C10) formed a transparent, self-standing, and injectable hydrogel. As the hydrogel contained both a protein and a lipid, a number of organic dyes were readily soluble in this hybrid matrix. Therefore, these gels were convenient for a variety of optical studies with embedded organic fluorophores. Our data showed that protein/lipid hydrogels can be tailored for fundamental spectroscopic studies such as light harvesting or white-light emission. Our results are described below.

### 2.1. Synthesis of BSA/C10 Hydrogels 

**Figure 1:**
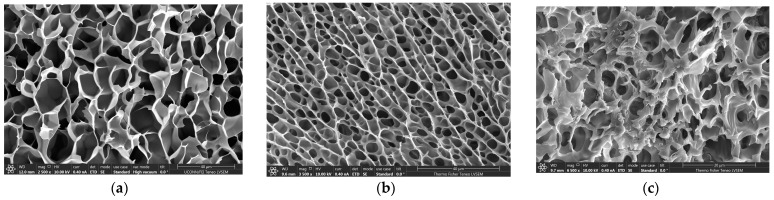
SEM images of BSA hydrogels at increasing concentrations of C10: (**a**) 100 mg/mL BSA with 0 mM C10; (**b**) 100 mg/mL BSA with 40 mM C10, which is lower than the critical micelle concentration of C10; and (**c**) 100 mg/mL BSA with 100 mM C10, which is higher than the critical micelle concentration of C10. The sizes of the individual cells within each gel were comparable and they decrease with an increase in C10 concentration. Gels contained about 90% water, and they were transparent, soft, and syringe-injectable.

Hydrogel formation as a function of C10 concentration was tested by preparing a series of hydrogels with increasing C10 concentrations (0, 20, 50, 80, and 100 mM) at a constant BSA concentration (~1.5 mM, 10% w/w). The samples containing C10 concentration above its critical micelle concertation (CMC 50 mM [22]) were opaque, as reported in the literature [20]. However, samples at higher C10 concentrations provided transparent hydrogels (Figure S1). BSA alone, without C10, provided only opaque gels and, thus, the presence of C10 was essential to obtain transparent gels for optical spectroscopy studies. Gel formation was tested by the standard inversion test and gel structure was examined by careful SEM analysis (Figure 1). 

The hydrogel samples were frozen in liquid nitrogen and water was removed under high vacuum to yield dry gels. SEM studies of thin sections of the dry gels clearly showed the porous scaffold of the BSA hydrogel (Figure 1a). The BSA hydrogel structure noted here was like that reported in the literature [20]. The openings in the BSA gel were less than 30 µm in size. This kind of structure is useful to isolate water pools within these spaces, and these spaces could be populated with C10 micelles. At C10 concentrations lower than the CMC of C10, the hydrogel showed a similar structure to that of BSA alone, but the pores were smaller in size and likely to be in the shape of tubes with clear edges (Figure 1b). When the C10 concentration was higher than its CMC, the hydrogel showed tubes and pore openings of less than 20 µm (Figure 1c). These morphologies were ideal for sequestering the donor–acceptor pairs within these hydrogels for optical studies.

To determine if the protein in the gel was loosely bound or if the free protein could be released into the aqueous phase, we examined the release of BSA into the aqueous phase as a function of time. The BSA/C10 hydrogel was equilibrated with deionized (DI) water and analyzed for free protein by a Bradford assay. In the absence of C10, no significant amount of protein was released, even after 34 h (Figure S2). With increasing concentrations of C10, though, there was a release of free protein up to nearly 15% and no significant further release was noted after 10 h. Thus, a fraction of the protein could be leached out of the hydrogel, but most of it could be retained in the gel matrix.

### 2.2. Circular Dichroism (CD) Studies

**Figure 2:**
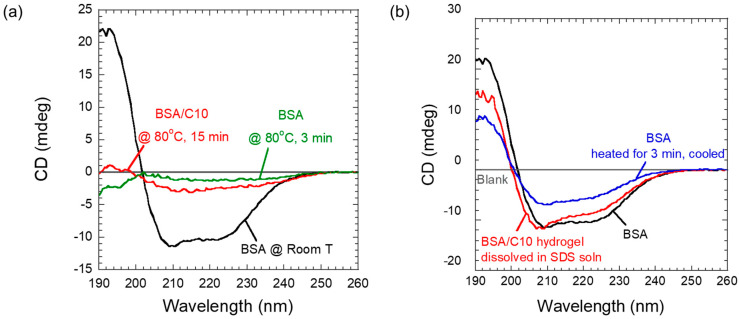
The circular dichroism (CD) spectra of BSA/C10 samples recorded at (**a**) 80 °C or (**b**) after heating to 80 °C followed by cooling to room temperature. The hydrogel sample was dissolved in 100 mg/mL SDS solution to match the BSA concentration in the gel the same as in the untreated BSA sample (2.5 µM).

The secondary structure of the protein was followed in CD studies. Upon heating to 80 °C, the BSA quickly denatured both in the absence of C10 or in the presence of C10 (100 mM) (Figure 2a). Even though the hydrogel resisted denaturation to a very small extent, it regained almost all of its original CD after cooling to room temperature (Figure 2b). Thus, above 80 °C, both BSA and BSA/C10 physical mixtures were likely to lose most of their CD signals, but on cooling, only the hydrogel regained its secondary structure almost completely (Figure 2b). For these studies, the hydrogel was dissolved in sodium dodecyl sulfate (SDS) solution after cooling to room temperature. The CD results demonstrated that C10 protects BSA from irreversible thermal denaturation or facilitates its refolding efficiently. Thus, we suspected that during the gel synthesis, the protein underwent denaturation, but on cooling to room temperature, the protein secondary structure was restored nearly to its native state. 

### 2.3. Gel Electrophoresis

**Figure 3:**
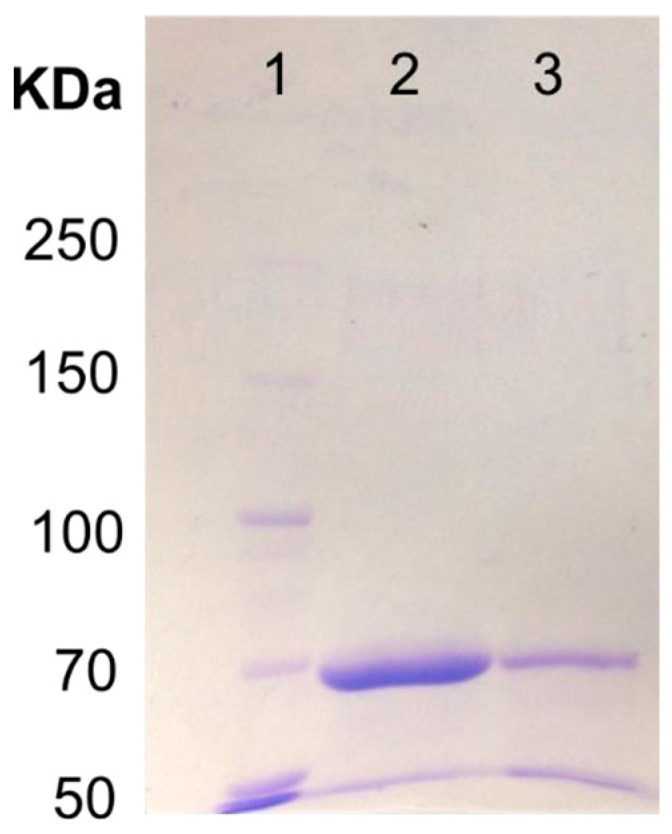
SDS–PAGE of the BSA/C10 hydrogel. Lane 1: molecular-weight marker with values shown in kDa; lane 2: BSA; lane 3: BSA/C10 hydrogel and gel dissolved in 100 mg/mL SDS solution for electrophoresis studies. Untreated BSA molar mass in lane 3 was around 65 kDa, as expected, which was evident in lane 3. All gels were run with 40 mM Tris acetate buffer at pH 7.

The status of any covalent crosslinking or degradation of the protein during the synthesis of the hydrogel was examined by SDS–PAGE. The SDS–PAGE (Figure 3) was run with untreated BSA, and the gel sample was dissolved in SDS solution to carry out the electrophoresis. The electrophoresis results showed that the molecular weight of BSA in the hydrogel (Lane 3) was around 65 kDa, the same as the untreated BSA (Lane 2), and no dimers, oligomers, or fragments of the protein were present in the gel. This ruled out any possibility of covalent crosslinking between the BSA molecules in the gels. Thus, the gels were formed by non-covalent assembly, paving the way for self-healing of the gels when they were mechanically damaged.

The BSA/C10 hydrogel was highly stable at room temperature, but when it was immersed in a large amount of water, the gel began to swell without falling apart. Moreover, the gel was stable in different pHs (from 1 to 10) for several days. The gel began to dissolve at pH 10 after 3 days. These results showed that clear BSA/C10 hydrogels could be synthesized via an easy and facile strategy, by simply heating the physical mixture without any reagents. This method did not harm the secondary structure of the protein, and the presence of C10, remarkably, protected the protein while promoting self-assembly into a transparent hydrogel that was suitable for optical studies. The resulting hydrogel was mechanically strong enough to be self-standing, but syringe-injectable. Next, we tested these hydrogels for potential applications as light-harvesting antennae and as white-light-emission coatings.

### 2.4. Binding Studies of Fluorescent Dyes to BSA 

Before assembling the four chromophores shown in Scheme 1 in the hydrogel to test for light-harvesting applications, we examined their affinity for BSA by fluorescence polarization anisotropy. Briefly, 10 µM solutions of each fluorescent dye were mixed with different concentrations of BSA. The fluorescence polarization anisotropy was measured and a non-linear regression was used to determine the dissociation constant (Figure S3, Table 1). All four dyes—rhodamine B (R), fluorescein (F), coumarin 540A (C), and Hoechst 55328 (A)—showed moderate affinity for BSA. These binding constants were in rough agreement with the literature reports [29], even though the experimental conditions used here were different from those reports.

Despite these moderate binding affinities of dyes for BSA, all four dyes were successfully embedded into the gel matrix by adding the dyes to the solution of BSA/C10 before heating. After heating, clear gels were formed successfully in each case. We postulated that the dyes could be embedded in these hydrogels at discrete binding sites, and that the gelation proceeded unimpeded in the presence of added dyes. 

### 2.5. Dye Leaching from the Hydrogels 

After the gels were formed in the presence of individual dyes, we tested for the leakage of dyes from the gels into the aqueous phase to understand their distribution between the gel matrix and the aqueous phase. The gels containing individual dyes were equilibrated with excess water and the amount of dye leaked into the supernatant was quantified as a function of equilibration time. 

Briefly, BSA/C10 hydrogel containing H, C, F, or R was placed in DI water, and supernatants were taken at different time intervals to determine the dye concentrations by absorbance spectroscopy and standard spectra (Figure S4). The percentage of the fluorescent dye that was retained in the hydrogel was calculated from these data and plotted as a function of equilibration time (Figure S5). 

The leaching studies demonstrated that protonated dyes under these conditions, such as H and C, were very well embedded in the gel and leached out slowly. On the other hand, neutral dyes, or dyes with some amount of anionic form (F, R), leaked out rapidly over a period of 10 h (Figure S5). The approximate leaching rates followed F > R > C >>> H, where H did not show any significant leaching. The slow leaching of H could have been due to the presence of multiple nitrogen atoms that could be protonated, contributing one or more positive charges that could interact favorably with the anionic lipid head groups. The leaching studies clearly showed that cationic dyes interacted strongly with the gel phase, while the anionic dyes favored the aqueous phase. Next, we examined the absorption, emission, and energy-transfer characteristics of these four dyes, first in the solution phase and, then, when embedded in the BSA/C10 hydrogel (Scheme 1).

### 2.6. Absorption and Emission Spectra of Dyes in Solution

Prior to the energy-transfer studies, we examined the spectral properties of the dyes in solution, in the presence of BSA or BSA and C10, to examine their photophysical properties in these media. In all samples containing H, C and F, [H] = 12.5 µM, [C540A] = 25 µM, [F] = 10 µM, [BSA] = 5 mg/mL, and [C10] = 5 mM. In samples containing rhodamine B, [R] = 20 µM, [BSA] = 1.25 mg/mL, and [C10] = 1.25 mM. For the emission spectral measurements, λ_excitation_ was 554 nm for R, 484 nm for F, 420 nm for C, and 350 nm for H. The spectral data are shown in Table 2.

**Figure 4:**
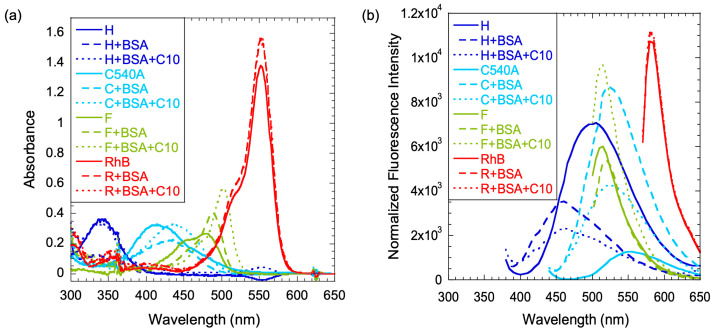
The absorbance spectra (**a**) and the emission spectra (**b**) of dyes in aqueous solution in the presence of BSA or both BSA and C10. Solutions remained clear at all concentrations tested here and there was no turbidity or precipitation.

For each dye, the absorbance and emission spectra were recorded in aqueous solution in the presence of BSA and in the presence of both BSA and C10 (Figure 4), while keeping the dye concentrations constant in each set. These samples were not heated and remained as solutions, without any precipitation or turbidity, throughout the study.

The absorbance spectrum of H with a maximum at 340 nm did not change with the addition of BSA or C10 when compared to the absorbance spectrum in the aqueous phase. The spectral properties of H were consistent with those noted in the literature [30]. In contrast, the peak maximum of C shifted by 23 nm to a longer wavelength upon binding to BSA, and the extinction coefficient decreased. With the addition of both BSA and C10, the absorbance peak stayed at the same position as it did with BSA, but the extinction coefficient was mostly restored to its original value. Thus, there was clear evidence that the microenvironment of C was subject to the presence of protein or protein and C10, which could be due to the key role of the protonation/deprotonation status of the single nitrogen present in its structure. The protonated form was expected to be more stable in a negatively charged microenvironment, and the spectral shifts were consistent with this line of interpretation [31].

In the case of F, the aqueous solution had a shoulder on the left of the peak in the range of 400–500 nm. Upon binding to BSA, the shoulder completely disappeared, and the peak position shifted to the red and the extinction coefficient increased. In the presence of both BSA and C10, the maximum peak position shifted further to the red, with an even higher extinction coefficient when compared to that recorded in the corresponding aqueous solution. The shoulder was from the anionic form of the dye, and it is likely that the negatively charged head groups of C10 destabilized the anionic form and the neutral form dominated [29]. In contrast to F, the absorbance spectrum of R did not show significant shifts by any of the additives, but had a slightly higher extinction coefficient in the presence of BSA or BSA/C10 solution. A lack of facile protonation/deprotonation sites on R could explain this behavior.

The emission spectra of the dyes also changed upon binding to BSA or to both BSA and C10. In the case of H, the emission shifted from 500 to 470 nm upon binding to BSA or BSA and C10. The intensity reduced in both cases when compared to the intensity recorded in water. 

The fluorescence of the coumarin dye C increased in the presence of BSA. The peak positions blue-shifted to 520 nm, while the intensity was enhanced ~7-fold. With the addition of both BSA and C10, the emission peak stayed at the same position as it did with BSA, but it had a higher emission intensity when compared to that in the aqueous phase. The higher fluorescence yields were welcome changes for energy harvesting, because they could potentially increase the amount of energy transferred to the acceptor dye, F. Another interesting feature was that the emission of C was blue-shifted so much that it resembled that of F and had greater overlap with the absorption spectrum of F, an important quantum mechanical parameter for facile FRET and for favorable positioning of the energy levels. 

In the case of F, the emission peaks did not shift to a significant extent when added to a solution of BSA or BSA and C10. However, the emission intensity of F increased dramatically in the presence of BSA/C10. This was presumably due to the conversion of the anionic form of the dye to the corresponding neutral form in the presence of a negatively charged environment of C10 [32]. Enhanced emission is an advantage for the energy-harvesting applications, as discussed above. 

The emission-peak positions of F did not change significantly upon the addition of either BSA or both BSA and C10. Interestingly, neither the absorption nor the emission spectra of R changed significantly with the addition of BSA or the BSA/C10 solution. The insensitivity of the properties of R to the media was consistent with our previous studies [10]. Next, we examined the energy transfer from donors to the corresponding acceptors to optimize dye concentrations for the light-harvesting studies, with all four dyes first in the solution phase, then in the gel phase. 

### 2.7. Fluorescence Energy Transfer Studies in BSA and BSA/C10 Solutions

**Figure 5:**
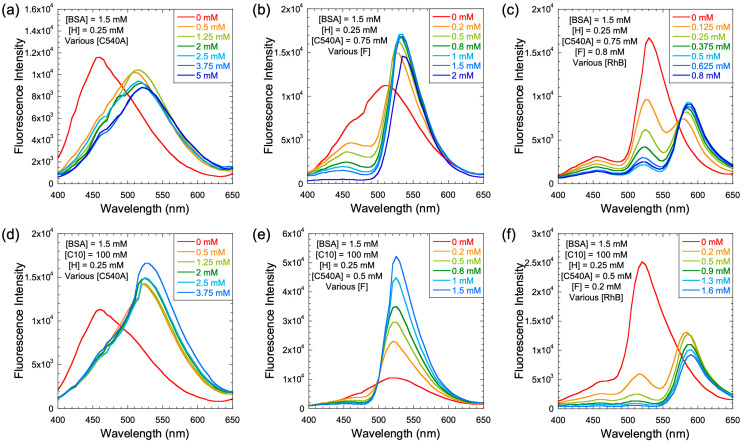
Fluorescence quenching of H emission by C (**a**,**d**), H, and C emission by F (**b**,**e**), and H, C, and F emission by R. The quenching studies were carried out in the solution phase in the presence of 100 mg/mL BSA (**a**–**c**), and with the presence of 100 mg/mL BSA and 100 mM C10 (**d**–**f**). Solutions remained clear at all concentrations tested here, and there was no turbidity or precipitation.

First, energy transfer was examined in the presence of a fixed concentration of BSA (1.5 mM), but increasing concentrations of one or more acceptors (Figure 5a–c). The concentration of H was kept constant at 0.25 mM, and the concentration of C was varied from 0 to 5 mM (Figure 5a). With C as low as 0.5 mM, all the emission from H was quenched and only the emission from C was observed at all concentrations of C, which indicated that the energy transfer from H to C was essentially complete.

Next, we examined the energy-transfer chain from H to C to F, at constant concentrations of BSA (1.56 mM), H (0.25 mM), and C (0.75 mM) without C10, while increasing the concentration of F from 0 to 2 mM. The addition of F gradually quenched the emission from C with the concomitant increase in F emission and at 2 mM F, and there was essentially complete quenching of the H/C emission (Figure 5b). Next, we examined the energy-transfer chain involving constant concentrations of H, C, and F as donors with increasing concentrations of R (0–0.8 mM) as the terminal acceptor (Figure 5c). Most of the emission from H, C, and F was quenched with a concomitant emission from R.

Next, donor–acceptor energy transfer was examined in the presence of constant concentrations of both BSA (1.5 mM) and C10 (100 mM) at increasing C concentrations in the solution phase. Essentially, at all concentrations of C, H emission was completely quenched as it was in the case of the BSA solution, with a corresponding emergence of emission from C (Figure 5d). However, in the case of H (0.25 mM), C (0.5 mM), and F (0 to 1.5 mM), the quenching of C emission was more rapid, with a concomitant increase in emission from F (Figure 5e), than it was with BSA alone. With the BSA/C10 solution, the emission from H or C was quenched completely with only 0.2 mM F, which was much more rapid than in the case of BSA alone. As with the BSA solution, the energy-transfer chain H to C to F to R yielded interesting results, where only 1.5 mM R was adequate to essentially wipe out all emission from the chain H/C/F and replace it with that of R (Figure 5f). These qualitative measurements were encouraging in designing the final energy transfer chain H/C/F/R and testing for the effect of C10 concentration on the energy-transfer cascade.

### 2.8. Effect of C10 on Energy-Transfer Cascade among All Four Dyes in Solution

**Figure 6:**
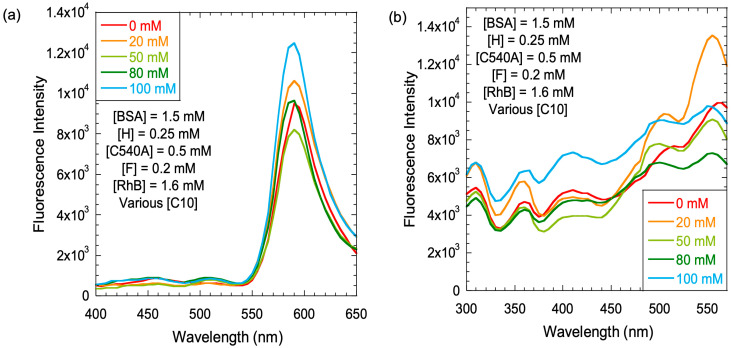
(**a**) The emission spectra and (**b**) excitation spectra of BSA/C10 solution at increasing concentrations of C10. [BSA] = 1.5 mM, [H] = 0.25 mM, [C] = 0.5 mM, [F] = 0.2 mM, and [R] = 1.6 mM. The emission spectra were recorded with the excitation at 350 nm, and the excitation spectra were recorded by monitoring emission at 590 nm. Solutions remained clear at all concentrations tested here, and there was no turbidity or precipitation.

Before examining the gels for energy cascade among the four dyes, one other requirement was to determine the effect of C10 concentration on the energy-transfer cascade. The concentration of BSA was kept constant at 1.5 mM, and the concentration of C10 was varied from 0 to 20 to 50 to 80 to 100 mM (Figure 6). Concentrations of all the dyes were kept constant. 

The emission spectra were comparable at all concentrations of C10, and R emission maxed out at 100 mM C10, with essentially complete quenching of H/C/F emission (Figure 6a). Such efficient energy transfer in the solution phase is rarely noted. Therefore, we decided to confirm the energy transfer from each of the three potential donors, and the emission at 590 nm was used to record the excitation spectra of the four-dye system (Figure 6b) at a constant BSA concentration, but with increasing C10 concentrations. 

The excitation spectra (590 nm monitoring) at all C10 concentrations clearly indicated excitation peaks corresponding to the absorption peaks of all four dyes. Even though there was an unexpected increase in R excitation intensity at 20 mM C10, this was strong evidence that the energy-transfer cascade was operational at all these concentrations of C10, from H to C to F to R.

**Figure 7:**
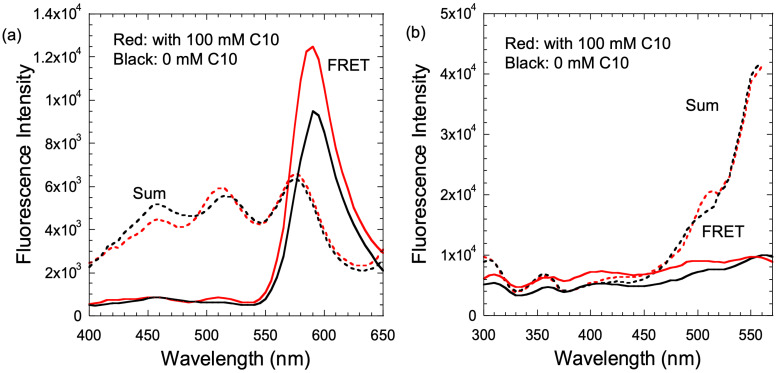
Comparison of (**a**) emission spectra and (**b**) excitation spectra with the sum of the spectra of each individual dye bound to BSA (with or without C10) with those of a sample containing all four dyes (FRET).

The success of FRET among the four dyes was also confirmed by comparing the sum of emission spectra of each individual dye with that of emission spectra containing all four dyes, BSA, and BSA/C10 solution (Figure 7a). The observed emission from the mixture of the four dyes had the characteristics of a 590 nm peak from R and the terminal acceptor, and was distinct from the sum of the four spectra of the individual components. Thus, the energy-transfer cascade to the terminal acceptor was again revealed. Similarly, the sum of the four excitation spectra of the individual dyes with the BSA/C10 solution indicated the peaks of all four dyes, which were distinct from that of the four-dye assembly (Figure 7b). Again, the energy transfer from each of the three donors to the terminal acceptor was well established in the presence of BSA/C10.

The energy cascade was interrupted if one of the four dyes was omitted from the assembly. The excitation wavelength was set at 350 nm for all samples, and the emission was scanned from 400 to 650 nm (Figure S6), while omitting one dye at a time. When H was absent, there was no emission corresponding to H, as expected, but emission from C/F and R was noted. In the absence of C, three separate peaks corresponding to emission from H, F, and R were noted. In the absence of F, there was emission from H/C and from R, which indicated energy transfer from H/C. Without R, the emission from F skyrocketed, further supporting the idea that R was the penultimate acceptor in the chain, but alternative pathways of the energy cascade to the terminal acceptor were also possible.

Having successfully established the energy-transfer cascade chain with the four different dyes by a variety of spectral studies in the solution phase, we then chose these dye concentrations, as well as BSA and C10 concentrations, to establish dye/BSA/C10 hydrogels for our next round of studies.

### 2.9. Cascade Singlet Energy Transfer in the Dye-Loaded Hydrogels

Hydrogels of BSA (1.5 mM) and C10 (100 mM) were prepared after dissolving appropriate amounts of the dyes in the liquid, prior to heating, to form the gel. Gels containing individual dyes, as well as all four dyes (H (0.25 mM), C (0.5 mM), F (0.2 mM), and R (1.6 mM)), were optically clear, free-standing, and injectable with a syringe. After mixing with the four dyes, the gel was pink-orange under room light, while it emitted bright-red fluorescence under UV light. 

**Figure 8:**
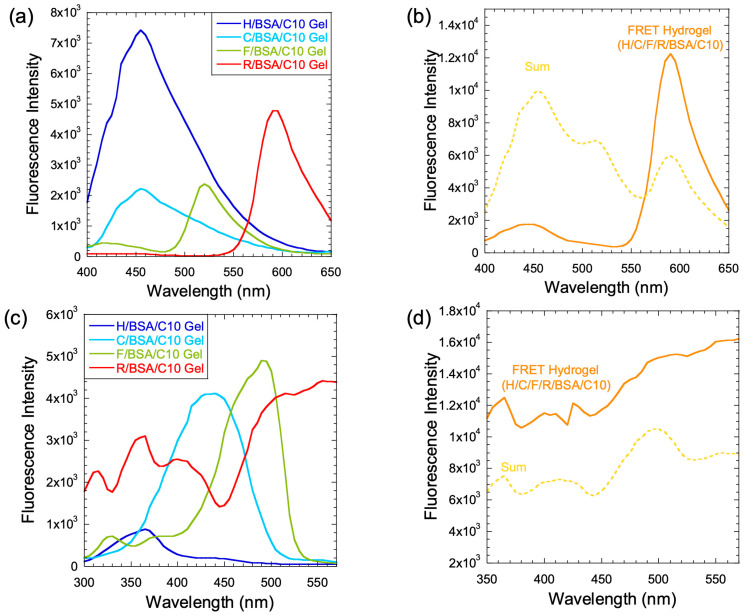
(**a**) The emission spectra of individual dyes embedded in BSA/C10 hydrogels. (**b**) The comparison of the sum of the four emission spectra of BSA/C10 hydrogels containing a single dye with that of BSA/C10 hydrogel containing all four dyes. (**c**) The excitation spectra of gels containing a single dye. (**d**) Overlay of the sum of the spectra of gels of single dyes with that containing all four dyes. The dye concentrations in the mixture were kept identical to the corresponding gels with a single dye.

The emission and excitation spectra of the gels containing individual dyes, as well as those of all four dyes, are compared in Figure 8. The emission spectra of the individual dyes embedded in the hydrogel indicated spectra (Figure 8a) that were comparable to those recorded with the corresponding aqueous solutions. Some minor changes were evident. Emissions of H and C were blue-shifted (450 and 500 nm) when compared to the emissions of the aqueous solutions, while the emission maximum of F and R were nearly the same (525 and 580 nm). These spectra were similar to those observed with the BSA/C10 solution.

The sum of emission spectra of BSA/C10 hydrogels containing each of the four dyes were overlaid with the spectra of emission from BSA/C10 hydrogel containing all four dyes (Figure 8b). The dominance of R emission in the four-dye spectrum demonstrated the energy-transfer cascade in the BSA/C10 gel. In the four-dye system, each of the four dyes were at identical concentrations, as they were in the corresponding single-dye-containing gels. Excitation spectra of each of the four dyes are shown in Figure 8c, with characteristic peaks of each of the individual dyes, as noted in the case of the BSA/C10 solution. Overlay of the four-dye excitation spectrum with the sum of the excitation spectra of the four individual dyes clearly indicated energy transfer among the four dyes (Figure 8d). These spectral data, although qualitative, clearly established that the energy-transfer cascade among the four dyes resulted in intense emission from the terminal acceptor, R. Encouraged by these fluorescence studies in the protein/lipid hydrogels, we proceeded to examine these gels for their use in the construction of white-light-emitting coatings.

### 2.10. White-Light-Emitting BSA/C10 Hydrogels 

White-light emission can be achieved by using blue-, green-, and red-emitting fluorophores, where the energy transfer among the excited states of the dyes is prevented or minimal [1]. This requirement is opposite to that needed for the energy-transfer cascade illustrated above, where dye-to-dye energy transfer was facilitated. At low dye concentrations, they could be dispersed in the gel phase, while keeping them sufficiently apart from each other [10,11]. Under these conditions, the energy transfer among the donor–acceptor pairs was impaired, and this could provide a simple approach to generating white-light emission gels. The compartmentalization of water pools in the BSA gels and the presence of both a protein and a lipid for dye-binding in the BSA/C10 hydrogel provided unique opportunities to test this idea. We chose a blue-emitting fluorophore, anthracene (A), a green-emitting F, and a red emitting R as the three-dye system to generate white light. As these molecules have distinct binding sites on BSA, at low dye concentrations, the energy transfer among the dyes was expected to be very small to none. Under the usual BSA/C10 gelation conditions, the addition of A, F, and R produced clear hydrogels. The composition of the three dyes was varied systematically to obtain white-light emission.

**Figure 9:**
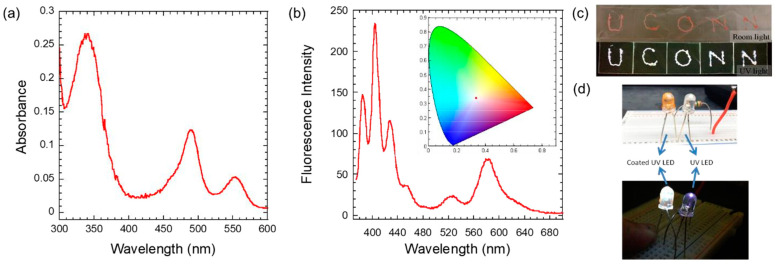
(**a**) The absorbance spectrum of BSA/C10 containing A, F, and R which indicated distinct peaks corresponding to the three fluorophores. (**b**) The emission spectrum of BSA/C10 containing A, F, and R, when excited at 365 nm. The inset shows the chromaticity plot from the observed emission spectrum, with CIE color coordinates of 0.336 and 0.339. (**c**) The soft hydrogel was applied by syringe on a glass slide in the desired shape. The letters appeared pink under room light, while they emitted white light when excited at 365 nm. (**d**) The gel converted blue light of a 365 nm UV LED to bright white light when applied as coating.

The absorbance spectrum BSA/C10 gel containing the three fluorophores and the emission spectrum with the excitation at 365 nm are shown in Figure 9. The absorbance spectrum of A was unusually broadened without its characteristic vibronic structure, but the spectra showed three separate peaks corresponding to those of A, F, and R. The emission spectrum of the gel (excitation at 365 nm) clearly indicated emission with characteristic peaks of the three fluorophores (Figure 9). Anthracene emission was, surprisingly, well-structured, despite its broad absorption spectrum, noted with the gel. The fluorescence spectra of F and R were as expected from the above studies.

The chromaticity coordinates of the emission were calculated to be 0.336 and 0.339—nearly pure white-light emission. The final concentrations for the successful white-light emission (excitation at 365 nm) were made from BSA (1.5 mM), C10 (100 mM), A (0.75 mM), F (0.075 mM), and R (0.058 mM). The molar ratio of these components (26:1724:13:1.3:1) indicated low concentrations of F and R, when compared to those used in the light-harvesting system discussed above. The low concentrations weakened any energy transfer between F and R, the donor–acceptor pair. Thus, the hydrogel is good in its ability to keep the chromophores from self-quenching or dye-aggregation.

As the hydrogel is syringe-injectable, the white-emitting gel was applied on the glass slide with a syringe to achieve the desired shapes (Figure 9c). The letters appeared to be pink under room light but appeared to be white under 365 nm UV light. The 365 nm UV LED emitted UV blue light without the gel coating, while the LED coated with the white-emitting hydrogel showed bright white-light emission (Figure 9d). Thus, the gels were versatile in the construction of either an energy cascade system with multi-step energy transfers to produce red emission or a shutting down of the energy transfer significantly to generate predominantly composite white-light emission from three different dyes.

## 3. Materials and Methods

### 3.1. Materials 

Bovine serum albumin (BSA) was purchased from Equitech-Bio, Inc. (Kerrville, TX, USA). Decanoic acid, sodium hydroxide, Hoechst 33258 (H), and fluorescein (F) were purchased from Sigma–Aldrich (Milwaukee, WI, USA). Coumarin 540A (C) was purchased from the Exciton Chemical Co. Inc. (Dayton, OH, USA). Rhodamine B (R) was purchased from the Eastman Kodak Company (Rochester, NY, USA). 

### 3.2. Synthesis of BSA/C10 Hydrogel 

The stock solution C10 (400 mM) was prepared by dissolving solid decanoic acid in deionized (DI) water, by adding small volumes of dilute solution of sodium hydroxide untill C10 completely dissolved. The stock solution of BSA (~3 mM) was prepared by dissolving 200 mg/mL BSA in DI water by stirring for few hours at room temperature. The concentration of BSA in the final solution was kept constant (100 mg/mL, 1.56 mM), while C10 concentration was varied from 0 to 100 mM. BSA and C10 solutions of specific compositions were mixed, then heated at 121 °C in an autoclave for 15 or 25 min to form hydrogels, as evidenced by the inversion test.

### 3.3. Scanning Electron Microscopy (SEM) 

Scanning electron microscopy images were carried out on a Teneo LVSEM (Thermo Fisher Scientific, Waltham MD, USA). BSA and BSA/C10 hydrogels were sliced with a razor blade and put into centrifuge tubes. The centrifuge tubes were punctured with a needle and dipped in liquid nitrogen before freeze-drying overnight. Once freeze-dried, the samples were further sliced and affixed to the Teneo LVSEM stub using carbon tape. Samples were taken for Au/Pd spin-coating (5 nm, 80% Au, 20% Pd) the next day, and imaged immediately.

### 3.4. Binding Studies of Dyes with BSA 

The binding constants of H, C, F, and R with BSA were determined by fluorescence polarization anisotropy. Briefly, a series of solutions containing 10 µM of fluorescent dye and different concentrations of BSA (1–128 µM) were prepared in DI water and equilibrated for at least an hour. Fluorescence anisotropy was measured with a Molecular Devices FlexStation 3 Microplate Reader (Molecular Devices, San Jose, CA, USA). The detector sensitivity was set as low, and an auto cutoff filter was used for the measurements. For R, λexcitation = 554 nm, λemission = 582 nm; for F, λexcitation = 484 nm, λemission = 513 nm; for C, λexcitation = 420 nm, λemission = 556 nm; for H, λexcitation = 350 nm, λemission = 460 nm. A simple equilibrium model was used to obtain the dissociation constant (K_d_) from the data, and Kaleidagraph was used for nonlinear curve-fitting, according to the following equation:

(1) $$r_{observed}=\{r_{F}+\left(r_{B}-r_{F}\right)\{{\left[\mathrm{BSA}\right]}_{T}/(K_{d}+{\left[\mathrm{BSA}\right]}_{T})\}$$

where r indicates the anisotropy, subscripts F and B indicate free and bound states, respectively, K_d_ indicates the dissociation constant, and [BSA]_T_ indicates the total concentration of BSA. 

### 3.5. Synthesis of Artificial Antenna System 

The artificial antenna system was obtained by adding the dyes H, C, F, and R. DI water was used to prepare the stock solutions of R (10 mM), F (10 mM), and H (5 mM), and dimethylformamide (DMF) was used to prepare the stock solution of C (25 mM). Solutions of these four dyes were added into a solution containing BSA and decanoic acid (C10). The mixture was then heated to form the hydrogel, as described. 

### 3.6. Absorbance and Fluorescence Measurements 

Absorbance spectra of the hydrogels placed on glass cover slips were collected using an HP 8453 diode array spectrophotometer from Agilent Technologies (Mendon, MA, USA). Steady-state fluorescence spectra were collected with a fluorescence spectrometer or a FlexStation 3 Microplate Reader (Molecular Devices, MA, USA). For the fluorescence spectrometer, the front phase accessory was used with slit widths set to 2.5 mm to collect the emission and excitation spectra of the hydrogels. During the measurements, glass cover slips were moved around and rotated to check for uniformity, and several vertical orientations at fixed incidence angle were used to obtain the average values.

### 3.7. Dynamic Light Scattering (DLS) 

The hydrodynamic size of BSA, decanoic acid micelles, and BSA/C10 complex were monitored by means of photon correlation spectroscopy with CoolBatch+ dynamic light-scattering apparatus (Varian Inc., Palo Alto, CA, USA) with a 10 × 10 mm^2^ cuvette and a 658 nm excitation laser source at a 90° geometry. Data collection was carried out at room temperature, 1 s response, 3 repetitions, and 100 accumulations. BSA and the nanoparticles were dissolved in 50 mM phosphate buffer (pH 8) and the solutions were filtered with 0.22 μm filters (polyvinylidene difluoride, PVDF, 13 mm, Restek Corporation, Bellefonte, PA, USA) before the measurements. Precision Elucidate v 1.1.0.9 and Precision Deconvolve v 5.5 (Varian Inc., Palo Alto, CA USA) from the manufacturer were used to collect and analyze the data. 

### 3.8. Circular Dichroism (CD) Spectroscopy

The retention of the secondary structure was tested by circular dichroism (CD). CD spectra were recorded on J-710 spectropolarimeter (JASCO, Okalahoma City, OK, USA). Samples (0.3 µM BSA) were created in 50 mM phosphate buffer (pH 8.0). Spectra were obtained using a 0.05 cm path length quartz cuvette from 260 nm to 190 nm. The sensitivity was set at 100 millidegrees and the data pitch was set at 0.5 nm, with continuous scanning mode, 50 nm min^−1^ scanning speed, 1 s response, 1.0 nm bandwidth, and three accumulations. The baseline was measured with 50 mM phosphate buffer (pH 8.0) and subtracted from each spectrum. 

### 3.9. Agarose Gel Electrophoresis 

Agarose gel (0.5%) was prepared by dissolving 125 mg agarose in 25 mL Tris acetate buffer (40 mM, pH 7.0) and microwaved for 1 min. Then, the agarose solution was poured onto the gel tray and cooled to room temperature. Samples were prepared by mixing 10 μL protein solution (1 mg/mL) with 10 μL of agarose loading buffer (50% *v*/*v* glycerol solution containing 0.01% *m*/*v* bromophenol blue). Then, 15 μL of each sample was loaded in each well (7.5 μg of protein/well). The agarose gel was run at 100 mV for 25 min with a Gibco BRL Model 200 (Billings, MT, USA) power supply. After running, the agarose gel was stained with 20% *v*/*v* acetic acid solution containing 0.003% *m*/*v* brilliant blue R-250 and destained with a 10% *v*/*v* acetic acid solution. 

### 3.10. Sodium Dodecyl Sulfate Polyacrylamide Gel Electrophoresis (SDS–PAGE) 

The gel contained a separating gel (7.5%) at the bottom and a stacking gel at the top. The samples were prepared by mixing 10 μL of protein solution (1 mg/mL) with 10 μL of loading buffer (2% SDS, 10% beta-mercaptoethanol (BME)) and heated in an 80 °C water bath for 5 min. Then, 15 μL of each sample was loaded in each well (7.5 μg of protein/well). The gel was run at 200 mV for 30 min with a Mini Protean Electrophoresis apparatus (Bio-Rad, Hercules, CA). Then, the gel was stained with stain I (10% *v*/*v* acetic acid, 10% *v*/*v* isopropanol, 0.003% *m*/*v* brilliant blue R-250) and stain II (20% *v*/*v* acetic acid, 0.003% *m*/*v* brilliant blue R-250) and destained with 10% *v*/*v* acetic-acid solution. The gels were scanned on an office scanner and digitized with NIH ImageJ software (V 1.52i., November, 2018, NIH, Bethesday, MA, USA).

### 3.11. Synthesis of White-Light Emitter 

Gels for white-light emission studies were made by using anthracene (A, blue), fluorescein (F, green), and rhodamine B (R, red) in the gel matrix ([BSA] = 100 mg/mL (~1.5 mM), [R] = 0.058 mM, [F] = 0.075 mM, and [A] = 0.75 mM.), prepared as above. White-light emission was obtained by systematically varying the dye concentrations and validated using CIE 1931 chromaticity coordinates. The chromaticity coordinates were calculated using MatLab 2013A and the open source “CIE Coordinate Calculator” by Prashant Patil [33].

## 4. Conclusions

Here, we presented an easy, quick, and facile strategy to form a unique protein/lipid hydrogel by heating a physical mixture of BSA and decanoic acid, with the goal of making a material that is ambidextrous, applicable for light-harvesting or for white-light emission. The gel can readily accommodate a variety of fluorophores, while retaining their photophysical characteristics, even with some improvements in some cases. These hydrogels use food-grade ingredients and are attractive for photophysical or other applications. The protein and the lipid, as well as the aqueous phase, provide distinctly different microenvironments to accommodate a variety of dyes to construct a light-harvesting system in which energy transfer from dye to dye is promoted. Alternatively, the energy transfer can be restricted to produce intense white light when excited in the blue. Thus, the diagonally opposite requirements of these two systems are easily met by the present hydrogel assembly, which simply depends on the dyes used and their concentrations. This flexibility is difficult to achieve in other competing systems. The physical flexibility of the injectable, but free-standing, hydrogel makes it a novel white-light emitter as a coating material for blue LEDs or light panels. It is a low-cost, easy-to-synthesize, biodegradable (when released into the environment), non-toxic, and soft material with potential for sustainability. Since the gels are syringe-injectable and non-toxic, we anticipate exciting energy applications.

■ Protein lipid hydrogels

Protein lipid hydrogels are a type of hydrogel that is composed of both proteins and lipids. Hydrogels are cross-linked networks of hydrophilic polymers that can absorb large amounts of water. Proteins are biomolecules that play a variety of roles in cells and tissues. Lipids are a class of organic molecules that are important for cell membrane structure and function.

The combination of proteins and lipids in hydrogels offers several advantages. Hydrogels can swell and shrink in response to changes in water content, which makes them ideal for applications such as drug delivery and tissue engineering. Proteins can provide additional functionality to hydrogels, such as the ability to bind to specific molecules or to degrade in response to certain stimuli.

■ Assay Methods

There are several different assay methods that can be used to study protein lipid hydrogels. Some common methods include:

- Gel permeation chromatography (GPC): GPC is a method for determining the size and molecular weight distribution of polymers. This method can be used to characterize the size and structure of protein lipid hydrogels.
- Dynamic light scattering (DLS): DLS is a method for measuring the size and movement of particles in solution. This method can be used to measure the size and swelling properties of protein lipid hydrogels.
- Water content: The water content of a hydrogel can be determined by measuring the weight of the hydrogel before and after it is fully hydrated.
- Mechanical properties: The mechanical properties of a hydrogel, such as its tensile strength and modulus, can be measured using a variety of methods.

■ Applications

Protein lipid hydrogels have several potential applications in a variety of fields, including:

- Drug delivery: Protein lipid hydrogels can be used to deliver drugs to specific tissues or cells. For example, hydrogels that are loaded with drugs can be injected into the body, where they swell and release the drugs at a controlled rate.
- Tissue engineering: Protein lipid hydrogels can be used to create artificial tissues and organs. For example, hydrogels that mimic the properties of the extracellular matrix can be used to grow cells in vitro.
- Biosensors: Protein lipid hydrogels can be used to create biosensors that can detect specific molecules. For example, hydrogels that are coated with antibodies can be used to detect the presence of antigens.

■ Conclusion

Protein lipid hydrogels are a promising new class of materials with a wide range of potential applications. The development of new methods for the synthesis and characterization of protein lipid hydrogels is an active area of research.

## Figures and Tables

**Table 1:** The binding constants with BSA extracted from fluorescence anisotropy studies.

	H	C	F	R
K_b_	1.1 × 10^5^ M^−1^	2.5 × 10^5^ M^−1^	1.4 × 10^5^ M^−1^	1.3 × 10^4^ M^−1^
K_d_	9 µM	4 µM	7 µM	76 µM

**Table 2:** Absorption and emission maxima of dyes ^a^.

Aqueous Solution	H	C	F	R
**Absorption maximum (nm)**	340	412	480, 450	550
**Emission maximum (nm)**	500	552	512	580
**BSA**				
**Absorption maximum (nm)**	340	435	490	550
**Relative extinction change**	1	0.68	1.66	1
**Emission maximum (nm)**	470	525	520	580
**Relative emission intensity**	0.5	7.2	0.92	1.1
**BSA + C10 solution**				
**Absorption maximum (nm)**	340	435	500	550
**Relative extinction change**	1	1	2.2	1.1
**Emission maximum (nm)**	470	525	512	580
**Relative emission intensity**	0.33	3.5	1.6	1

^a^ Red numbers indicate a red shift of the spectral maximum, while blue color represents a blue shift the spectral maximum, in relation to the respective spectra in the controls.

## Data Availability

Data available in private archive only. Contact the corresponding author if access to data is required.

## Supplementary Materials

The following supporting information can be downloaded at: https://www.mdpi.com/article/10.3390/molecules28166028/s1. Binding data, absorption and emission spectra, gel photographs and excitation spectra are available as Supplementary Files (Figures S1–S6).
