# Supplementary material for: Non-Covalent Assembly of Multiple Fluorophores in Edible Protein/Lipid Hydrogels for Applications in Multi-Step Light Harvesting and White-Light Emission
Source: Molecules. 2023 Aug 12;28(16):6028. doi: 10.3390/molecules28166028 (PMC10458872; doi:10.3390/molecules28166028)
Supplement: Supplementary file 1 [file molecules-28-06028-s001.zip › molecules-2455921-supplementary.pdf]

Non-covalent Assembly of Multiple Fluorophores in Edible Protein/Lipid Hydrogels for Applications in Multi-step Light-harvesting and White-light Emission

Jingwen Ding and Challa V. Kumar*
Department of Chemistry, University of Connecticut, Storrs CT 06269 USA
*Corresponding author: challa.kumar@uconn.edu; (US) 860-486-3213

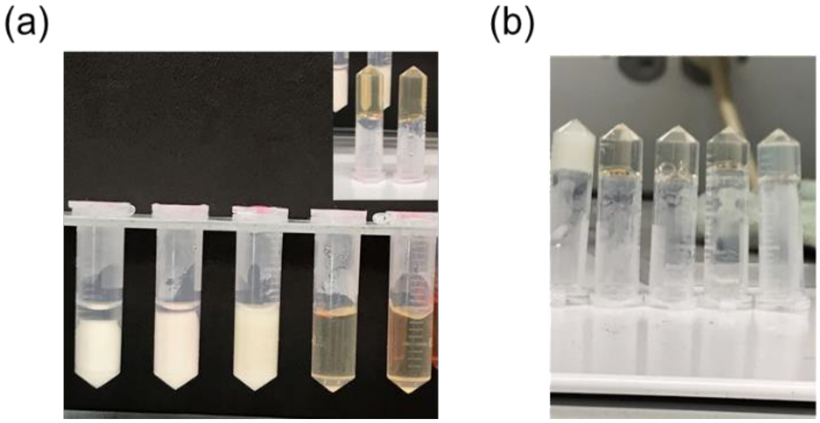

Figure S1. Photographs of BSA/C10 hydrogels made at increasing concentrations of C10, on heating in an autoclave at 121 ˚C for (a) 25 minutes and (b) 15 minutes. From left to right in both panels, the concentration of C10 was 0, 20, 50, 80, or 100 mM while BSA concentration was kept constant at 100 mg/mL. The insert in (a) and (b) shows the inversion test and confirms gelation in all samples. The presence of at least 20 mM C10 and 15-minute heating time produced transparent hydrogels. In the absence of C10, most of the gels were opaque to the naked eye. Thus, C10 was essential to produce robust BSA hydrogels and gels produced at lower temperatures or concentrations were softer and not viable for easy handling.

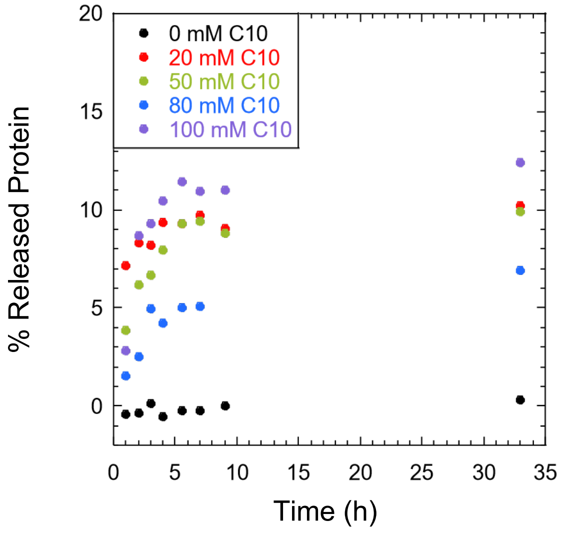

Figure S2. Protein release studies of the hydrogels at increasing concentrations of C10. The concentration of BSA was kept constant at 100 mg/mL, while C10 concentration has been varied from 0 to 20, 50, 80 or 100 mM. 300 μL of each hydrogel was carefully

injected to the bottom of 2 mL of water in a vial, and the amount of protein released into the supernatant has been determined by Bradford assay, as a function of time. C10 facilitated protein leakage from the gel suggesting the non-covalent binding of the two components.

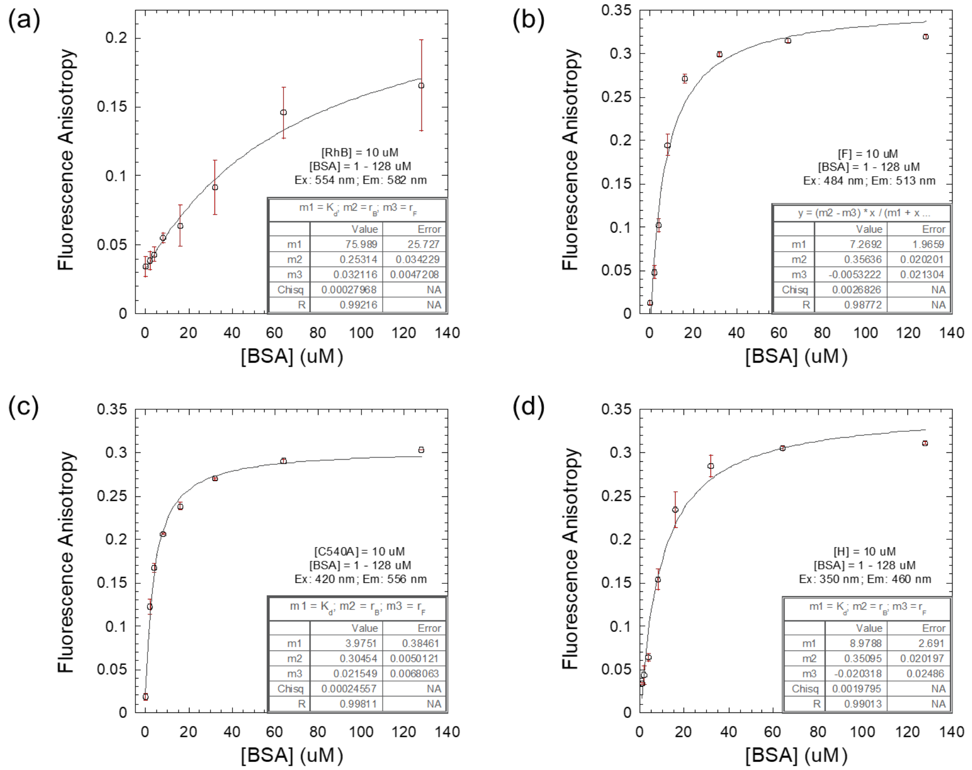

(a) $m1 = K_d$; $m2 = r_B$; $m3 = r_F$

	Value	Error
m1	75.989	25.727
m2	0.25314	0.034229
m3	0.032116	0.0047208
Chisq	0.00027968	NA
R	0.99216	NA

(b) y = (m2 - m3) * x / (m1 + x ...

	Value	Error
m1	7.2692	1.9659
m2	0.35636	0.020201
m3	-0.0053222	0.021304
Chisq	0.0026826	NA
R	0.98772	NA

(c) $m1 = K_d$; $m2 = r_B$; $m3 = r_F$

	Value	Error
m1	3.9751	0.38461
m2	0.30454	0.0050121
m3	0.021549	0.0068063
Chisq	0.00024557	NA
R	0.99811	NA

(d) $m1 = K_d$; $m2 = r_B$; $m3 = r_F$

	Value	Error
m1	8.9788	2.691
m2	0.35095	0.020197
m3	-0.020318	0.02486
Chisq	0.0019795	NA
R	0.99013	NA

Figure S3. Fluorescence polarization anisotropy of each fluorescent dye with BSA, as (a) Rhodamine B, (b) fluorescein, (c) coumarin 540A, and (d) Hoechst 55328. The concentration of each dye was kept as 10 μM, and the concentration of BSA was varied from 1 to 128 μM. The dissociation constants were calculated as m1 in each graph.

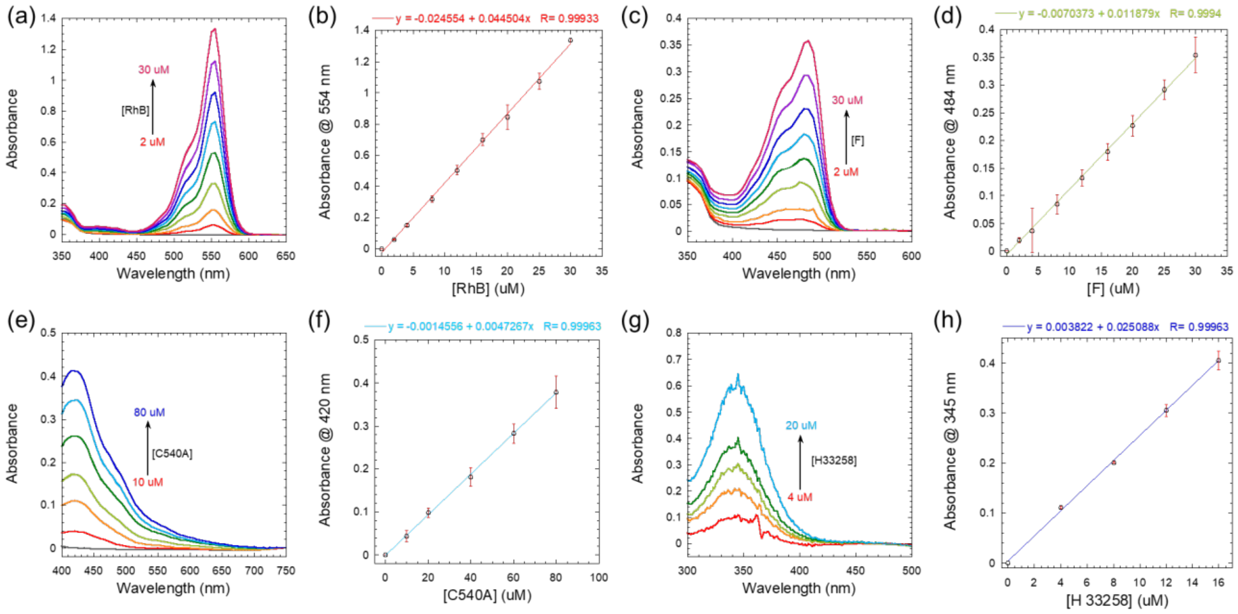

Figure S4. The absorbance spectra of (a) rhodamine B, (c) fluorescein, (e) coumarin 540A, and (g) Hoechst at increasing concentrations in water. The maximum absorbance peak intensity was then plotted versus the concentration of the dye to obtain the standard curves of (b) rhodamine B, (d) fluorescein, (f) coumarin 540A, and (h) Hoechst.

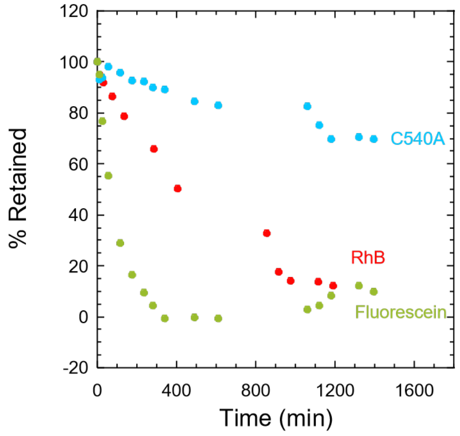

Figure S5. Leaching of fluorescent dyes from BSA/C10 hydrogels as a function of time. Although there was some scatter in data around 800 minutes due to a systematic error, error bars on the data are too small compared to the size of the data points.

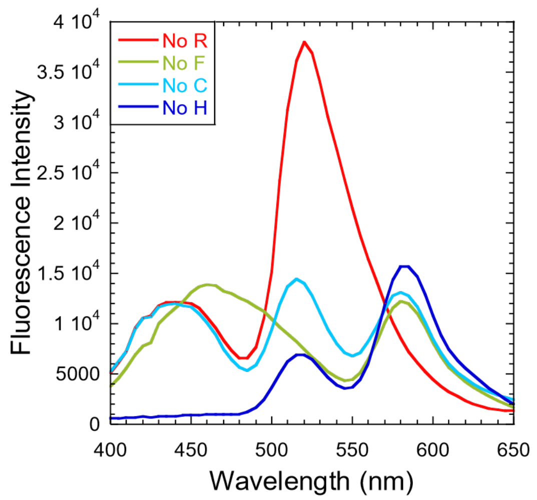

Figure S6. The emission spectra of BSA/C10 solutions containing 3 dyes, while omitting one of the four dyes. [BSA] = 1.5 mM, [H] = 0.25 mM, [C540A] = 0.5 mM, [F] = 0.2 mM, and [RhB] = 1.6 mM. The excitation wavelength was at 350 nm, and the emission scanned from 400 to 650 nm, FRET in gel phase.
